# Effect of *Cardinium* Infection on the Probing Behavior of *Bemisia tabaci* (Hemiptera: Aleyrodidae) MED

**DOI:** 10.1093/jisesa/ieab040

**Published:** 2021-06-19

**Authors:** Liu Ying, Liu Baiming, Li Hongran, Ding Tianbo, Tao Yunli, Chu Dong

**Affiliations:** 1 Key Lab of Integrated Crop Pest Management of Shandong Province, College of Plant Health and Medicine, Qingdao Agricultural University, Qingdao 266109, China; 2 Institute of Plant Protection, Tianjin Academy of Agricultural Sciences, Tianjin 300112, China; 3 College of Plant Protection, Nanjing Agricultural University, Nanjing 210095, China

**Keywords:** eletropenetrography, phloem sap ingestion, whitefly, endosymbiont, insect behavior

## Abstract

Facultative endosymbionts can affect the growth, physiology, and behavior of their arthropod hosts. There are several endosymbionts in the invasive whitefly *Bemisia tabaci* Mediterranean (MED, Q biotype) that influence host fitness by altering stylet probing behavior. We investigated the probing behavior of *B. tabaci* MED infected with the facultative endosymbiont *Candidatus* Cardinium hertigii (*Cardinium* (Sphingobacteriales: Flexibacteraceae)). We generated genetically similar *Cardinium*-infected (C^*+^) and uninfected (C^-^) clonal sublines and analyzed the probing behavior of newly emerged adult on cotton (Malvales: Malvaceae), *Gossypium hirsutum* L., using electropenetrography (EPG). The C^-^ subline demonstrated a longer duration of E2 (2.81-fold) and more events of E2 (2.22-fold) than the C^*+^ subline, indicating a greater level of sustained ingestion of plant phloem. These findings provide insight into the fitness costs (fitness of a particular genotype is lower than the average fitness of the population) of the *Cardinium*-infected *B. tabaci*.

Symbiotic bacteria can impact host nutrition, survival, reproduction, pesticide resistance, and adaptability (Kapantaidaki et al. 2015). A strong association can exist between a facultative endosymbiont and the fitness of their arthropod host (Simon et al. 2003, Miller et al. 2010). In phloem sap-sucking insects, endosymbionts can influence host aphid fitness by altering their stylet probing behavior (Lei et al. 2010a; Alvarez et al. 2010; Jiang et al. 2010a,b). *Wolbachia* infection can negatively influence mosquito blood-ingesting ability (Turley et al. 2009, Moreira et al. 2009). The destruction of symbiotic bacteria is detrimental to probing behavior of the pea aphid, *Acyrthosiphon pisum* Mordvilko (Hemiptera: Aphididae) (Wilkinson et al. 1995). Understanding the mechanisms of these effects on probing behavior may aid in future pest management of whiteflies in crops.

The sweet potato whitefly, *Bemisia tabaci* (Gennadius) (Hemiptera: Aleyrodidae), is an invasive species that causes substantial losses in crops worldwide. It damages tomato, cucumber, eggplant, and collards (Qiu et al. 2005, Dinsdale et al. 2010, Hu et al. 2020). This species complex contains at least 44 cryptic species, and more than 1,000 host plants have been recorded (Abd-Rabou and Simmons 2010, Kanakala and Ghanim 2019). Among them, *B. tabaci* Mediterranean (MED, also known as *B. tabaci* biotype Q) has replaced *B. tabaci* Middle East Asia Minor 1 (MEAM1, also known as *B. tabaci* biotype B), which was first introduced into China in 2003. It has now become the dominant species throughout China (Chu et al. 2006, 2010; Pan et al. 2011; Li et al. 2017).

*Bemisia tabaci* generally contains facultative endosymbionts, which vary among the cryptic species and geographical areas. Endosymbionts are classified as primary symbionts (P-symbionts) and secondary symbionts (S-symbionts) and each has an important ecological and evolutionary role. The P-symbiont, *Portiera* is confined to bacteriocytes and is essential for the survival and development of the host insect (Baumann 2005). Several S-symbionts, such as *Wolbachia*, *Cardinium* (Sphingobacteriales: Flexibacteraceae), *Hamiltonella*, *Fritschea*, *Arsenophonus*, and *Rickettsia*, can manipulate various physiological characteristics of their hosts, and differ among cryptic species (Gottlieb et al. 2006, Chiel et al. 2007, Gueguen et al. 2010).

*Candidatus* Cardinium hertigii (hereafter referred to as *Cardinium*) is a maternally inherited, facultative symbiont in *B. tabaci* MED (Zchori-Fein and Perlman 2004). It is generally known for improving host fitness (Weeks et al. 2004, Kenyon and Hunter 2007, White et al. 2011). Our long-term field surveys in Shandong Province showed that a low level of *Cardinium* infection (<17.3%) occurs in *B. tabaci* MED. *Cardinium*-infected (C^+^) has less competitive ability and fitness than *Cardinium*-uninfected (C^-^) (unpublished data; Fang et al. 2014). Therefore, after eliminating the genetic background, we hypothesized that the adverse effects of *Cardinium* on the fitness of its host may be associated with alterations in stylet probing behavior.

Electropenetrography (EPG) is an electronic technique used to record the real-time probing behavior of phloem sap-ingesting insects, such as *Aphis fabae* Scopoli (Hemiptera: Aphididae), represented by waveforms (Tjallingii 1978, 1985; Tjallingii and Esch 1993; Prado and Tjallingii 1997). This technology has been used to monitor whitefly probing behaviors (Zhang et al. 2016; Chesnais and Mauck 2018). Jiang et al. (1999) categorized the basic waveforms and found differences in probing behaviors among *B. tabaci* biotypes. Zhang et al. (2016) showed that the *Cardinium*-uninfected (C^-^) and *Cardinium*-infected (C^+^) sublines of *B. tabaci* MED showed differences in probing behaviors. The total duration of probes (using the waveform naming convention of Tjallingii (1978); also known as mean Probing Duration per Insect [PDI] in the convention of Backus et al. (2007) and the total duration of the pathway waveform (C waveform) of the C^+^ subline were significantly higher than those of the C^-^ subline, and the duration of NP (non-probing waveform) after the 1st E of the C^+^ subline was significantly lower than that of the C^-^ subline (Zhang et al. 2016). This indicates an association between *Cardinium* and alterations in *B. tabaci* MED’s probing behavior.

We hypothesized, based on the research of Zhang et al. (2016), that *Cardinium* is harmful to the probing behavior of *B. tabaci*. Therefore, we studied the differences in probing behaviors between *Cardinium*-uninfected (C^-^) and *Cardinium*-infected (C*^+^) *B. tabaci* MED sublines, after eliminating the influence of different genetic backgrounds. We reared different pairs of C^-^ and C^+^ whiteflies in the laboratory, as well as an introgression subline of *Cardinium*-infected (C^*+^) whiteflies from Shandong Province. The probing behaviors of C^-^ and C^*+^ whiteflies on cotton plants were examined using EPG, because cotton is an important crop in Shandong Province. Our results supported our hypothesis. They provide information about the mechanisms involved in the fitness decrease in *Cardinium*-infected *B. tabaci* MED’s, and provide a reference for studying the interaction of plants and insects.

## Materials and Methods

### Experimental Cotton Plant Cultivation

Cotton, *Gossypium hirsutum* L., cv. ‘Lu-Mian-Yan 21’ produced at the Shandong Cotton Research Center (Jinan, Shandong, China), was used in the probing behavior study. The plants (2–3 true leaf stage) were grown in isolated screen cages (200 mesh [40 × 30 × 50 cm], 5–6 cotton plants in each cage) in plant rearing chambers under controlled environmental conditions (27 ± 1°C; 16:8 [L:D]; 60 ± 5% relative humidity [RH]) and were watered every 5–7 d.

### Establishment of C^-^ and C^+^ Whitefly Sublines

*Bemisia tabaci* MED whiteflies were initially collected from Shouguang, Shandong Province in August 2017. These whiteflies were reared on cotton plants in isolated screen cages in climatic chambers under controlled conditions (27 ± 1°C; L: D [L:D]; 60 ± 5% relative humidity, RH). Approximately 200 field-collected whiteflies were transferred onto a cotton seedling (Li et al. 2012). *Bemisia tabaci* was reared in our laboratory self-made insect-rearing cups (each cup had one cotton seedling, one female and one male *B. tabaci*) until *B. tabaci* had laid eggs. Then the progeny were collected, and the parent and five offspring were frozen and scored for *Cardinium* infection using PCR. The primer used to detect *Cardinium* was CFB-F (5′-GCGGTGTAAAATGAGCGTG-3′) and CFB-R (5′-ACCTMTTCTTAACTCAAGCCT-3′) (Weeks et al. 2003). These were used to amplify ≈ 450 bp. Each PCR run consisted of: the first cycle at 95°C for 5 min, followed by 35 cycles at 94°C for 1 min, 58°C for 1 min, 72°C for 1 min, and final extension of 5 min at 72°C. We divided the remaining *B. tabaci* into *Cardinium*-uninfected (C^-^) and *Cardinium*-infected (C^+^), and reared them in different mesh cages. In this way, C^-^ and C^+^ sublines were established from Shandong origins. Tests proved that all C^-^ and C^+^ sublines were infected with the obligate nutritional symbiont *Portiera* and the facultative symbionts *Rickettsia* and *Hamiltonella* (unpublished data). The *Cardinium* of each subline was monitored every 30 d by sampling 20 adults as described previously (Fang et al. 2014).

### Introgression of C^-^ and C^+^ Whitefly Sublines

For the collected C^-^ and C^+^ sublines, an introgression backcrossing scheme was conducted in the laboratory to minimize genetic differences and homogenize the nuclear backgrounds of both whitefly sublines in the hosts (Brelsfoard et al. 2008). We introgressed the C^-^ subline nuclear background into the C^+^ subline over six generations to obtain C^*+^ and C^-^ sublines that shared > 98% of the nuclear alleles (Himler et al. 2011). The introgression process and flowchart were similar to those of Li et al. (2018). Whitefly colonies of both C^*+^ and C^-^ sublines were maintained on cotton plants in net cages in separate chambers in a glasshouse under the same conditions, without exposure to chemical insecticides. Whiteflies of the same age were collected for experiments within 24 h after becoming newly emerged adult. Whiteflies were then wired and prepared for EPG recordings.

### Endosymbiotic Bacterial Detection

*Bemisia tabaci* MED females were examined under a microscope (Nikon, Shanghai, China) and stored in a 0. 2-ml centrifuge tube at −20°C. In order to unify the experiment, this experiment uniformly uses the female *B. tabaci*. After that, *B. tabaci* DNA was extracted and used as a template for PCR amplification to detect the endosymbiotic bacteria (Chu et al. 2005). Sterile water was used as the negative control, and the DNA of *B. tabaci* MED infected with endosymbiotic bacteria was used as the positive control. The primers and PCR used to detect *Cardinium* were the same as above. The PCR products were electrophoresed on an agarose gel (1.0%) with negative and positive controls and visualized using a gel imager (Tanon, Shanghai, China). The C^*+^ subline had clear bands, while the C^-^ subline had no bands.

### EPG Recording

**Fig. 1.**
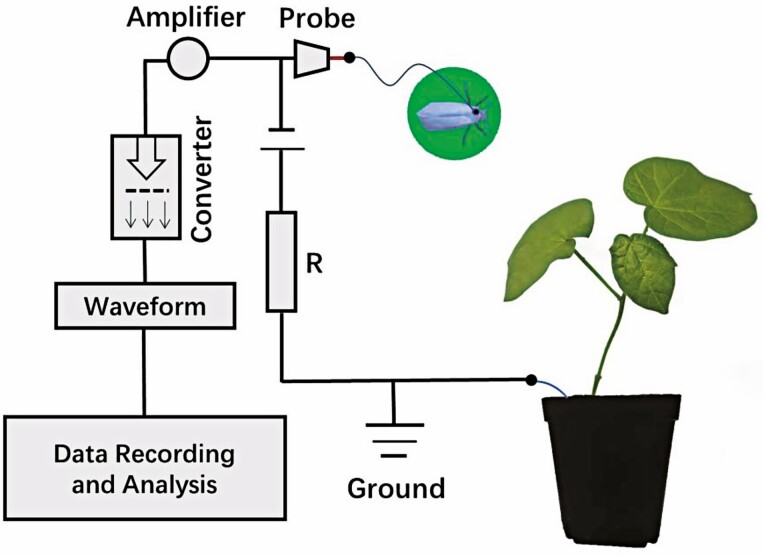
Schematic representation of electrical penetration graph (EPG) system.

The stylet probing behaviors of whiteflies on cotton plants were characterized by EPG using a direct-current electropenetrograph (DC-EPG) with a 10^9^-Ohm input resistance system (EPG Systems Wageningen University, Netherlands). Twenty-eight successful EPG recordings were obtained for each of the C^*+^ and C^-^ sublines of *B. tabaci* MED. Cotton plants, insects, and EPG head stage amplifiers were placed into electrically grounded Faraday cages to shield the setup from external electrical noise. Before recording, a newly emerged female (exactly 24 h old) whitefly, was picked randomly either from the C^*+^ or C^-^ population, starved for 20 min, and placed in a frozen glass dish (4 cm in diameter). Then, a 1.5-cm long gold wire (12.5 μm diameter) was quickly attached to the whitefly dorsum by applying a droplet of water-based silver glue for about 10 s until the glue dried. The wired whitefly was connected to the DC Giga-8 head-stage amplifier, and then placed on the abaxial side of a cotton leaf. EPG waveform signals were digitized with a DI710-UL analog-to-digital converter (DATAQ Instruments, Akron, OH), and waveforms were acquired using software the PROBE 3.4. The EPG was adjusted before each experimental run to make the signal fit within the voltage range on the computer screen. Each *B. tabaci* was provided one cotton plant to record continuously with EPG for 6 h for each recording. The experimental design is shown in Fig. 1. All experiments were carried out under controlled conditions (27 ± 1°C; 16:8 [L:D]; 60 ± 5% relative humidity, RH).

### EPG Waveforms and Parameters

The EPG waveforms were categorized as previously described (Jiang et al. 2010a,b). Five types of waveforms, including non-probing (NP), intercellular stylet pathway (C), potential drop (pd) (brief intracellular punctures), and phloem phases E1 (salivation into phloem) and E2 (phloem sap ingestion). The waveforms F and G were rare, and therefore grouped into the pathway waveform C. F represents mechanical derailment of the stylets, inside or outside of a cell, and G is the wave that represents active ingestion from the xylem. The time from the beginning to the end of each waveform event was manually marked and all event durations were then exported using PROBE. Thereafter, each recording was divided into two categories for behavioral analysis: non-phloem behaviors (NP, C, and pd) and phloem behaviors (E1 and E2).

### Statistical Analysis

Data were analyzed by the Shapiro–Wilk test (SPSS 13.0, Inc. I1 Chicago) to certify that they followed a normal distribution. If the data were normally distributed, an independent sample t-test was used to analyze the differences. A Mann–Whitney *U* test analysis was used if the data did not conform to normal distribution. The variables were selected based on their biological significance (significance level, α = 0.05). These variables were conventional variables recorded in the EPG of piercing and sucking insects because they reflect the entire probing process of insects.

## Results

**Fig. 2.**
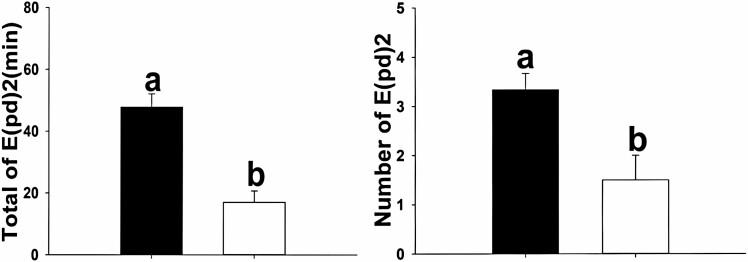
Phloem EPG parameters of *Cardinium*-uninfected (C^-^, dark bars) and *Cardinium*-infected (C^*+^, white bars) *B. tabaci* MED. Data are mean ± SD (*n* = 28). Different lowercase letters above the bars indicate a significant difference between C^-^ and C^*+^ (*P* < 0.05).

The *Cardinium-*uninfected *B. tabaci* MED (C^-^) subline was more conducive to probing than was the *Cardinium*-infected subline (C^*+^) (Fig. 2). However, no significant differences in probing behaviors were observed between the *Cardinium-*uninfected (C^-^) and the *Cardinium*-infected (C^*+^) *B. tabaci* MED sublines in the non-phloem phase (10 EPG variables) on cotton (*P* > 0.05; Table 1).

**Table 1.** Non-phloem EPG parameters for monitoring the feeding behavior of *Cardinium*-uninfected (C^-^) and *Cardinium*-infected (C^*+^) *B. tabaci* MED on cotton

	Whitefly strain		
Parameters	C^-^	C^*+^	*P*
Total duration of probes (min)	257.29 ± 12.66	269.66 ± 9.44	0.437
Total number of probe/*n*	150.04 ± 10.09	152.14 ± 8.79	0.875
Average time of probe (min)	2.01 ± 0.20	1.92 ± 0.12	0.577
Total duration of np (min)	95.19 ± 13.19	98.76 ± 14.95	0.909
Total duration of C (min)	250.76 ± 12.32	260.46 ± 9.84	0.541
Duration of first probe (min)	1.33 ± 0.29	1.84 ± 0.60	0.670
Probe duration before first E (min)	15.81 ± 4.42	27.51 ± 9.53	0.284
Number of probes before first E	9.08 ± 1.95	18.50 ± 6.01	0.166
Duration of np after first E (min)	95.22 ± 23.20	90.58 ± 18.93	0.883
Time to first E2 from first probe (min)	52.30 ± 21.14	99.98 ± 3.59	0.180

*indicates significant differences (*P* < 0.05) between the two sublines (Mean ± SD; *n* = 28).

Six additional variables were analyzed to investigate the influence of *Cardinium* on probing behavior during the phloem phase of *B. tabaci* MED on cotton (Table 2, Fig. 2). Differences between the C^*+^ and C^-^ sublines were observed in two of the six phloem variables evaluated. The total duration of E2 (also known as the mean Waveform Duration per Insect [WDI] for E2) and the number of E2 (also known as the mean Number of Waveform Events per 201 Insect [NWEI] for E2) (Backus et al. 2007) were the only variables that were significantly different between the C^*+^ and C^-^ sublines (*P* < 0.05; Fig. 2). The total of E2 duration and the number of events of E2 were 2.81 and 2.22 times higher in the C^-^ subline than in the C^*+^ subline, respectively.

**Table 2.** Phloem EPG parameters for monitoring the feeding behavior of *Cardinium*-uninfected (C^-^) and *Cardinium*-infected (C^*+^) *B. tabaci* MED on cotton

	Whitefly sublines		
Parameters	C^-^	C^*+^	*P*
Total duration of E1 (min)	1.02 ± 0.26	2.63 ± 0.99	0.186
Number of E1	8.38 ± 1.84	11.00 ± 1.43	0.446
Mean duration of E1 (min)	0.11 ± 0.01	0.30 ± 0.13	0.077
Mean duration of E2 (min)	14.61 ± 1.91	11.85 ± 1.51	0.388

*indicates significant differences (*P* < 0.05) between the two sublines (Mean ± SD; *n* = 28).

## Discussion

The EPG technique helps analyze the otherwise-invisible insect stylet penetration activities within plant tissues. EPG technology has been used to study the behavior or physiology of sap-sucking insects (mainly Hemiptera) on host plants (Backus et al. 2019, 2020). Whitefly stylet probing has characteristic appearances that are similar to waveforms of aphids, especially the pathway phase and phloem ingestion (Jing et al. 2013). Luo et al. (2005) found that, while EPG waveforms for some hemipterans can be quite diverse, they are stable for sternorrhynchan sap-sucking insects such as aphids and whiteflies. Therefore, in this experiment, we used the EPG technique to analyze the effect of *Cardinium* on the probing behavior of *B. tabaci* MED after eliminating the genetic background of *B. tabaci*.

*Bemisia tabaci* MED ingests phloem sap to obtain nutrients, such as amino acids and sugars (Jiang and Walker 2010b, He et al. 2011). The sugars provide energy for growth and development (Nardone et al. 2013, Detrain and Prieur 2014). In EPG recordings, phloem ingestion variables directly reflect the adaptive stylet probing behavior of sternorrhynchan insects. Prado and Tjallingii (1997) showed that the duration of phloem ingestion directly affects the number of insects ingesting in the phloem. Our EPG recordings revealed that the only difference between whiteflies, with or without endosymbionts, was in their ability to ingest phloem sap. Both the total duration of E2 (also known as WDI for E2) and the number of E2 events (also known as NWE1 for E2) of the *Cardinium*-uninfected (C^-^) subline in the phloem phase were longer than those of the *Cardinium*-infected (C^*+^) subline (Fig. 2), indicating modification of whitefly behavior by the secondary endosymbiont bacteria. Endosymbiont presence apparently decreased phloem ingestion. Less E2 means that the insect had decreased phloem ingestion, less nutrition, and possibly reduced development and reproduction. Non-phloem variables indicate the probing process before or after *B. tabaci* MED reaches the phloem. *B. tabaci* MED often requires multiple probes to reach the phloem (Lei et al. 2001, 2010b). Prolonged probing consumes more energy to find a suitable feeding site and results in lower ingestion efficiency. In this study, the probing behavior of C^*+^ and C^-^ sublines on cotton was not significantly different in the non-phloem phase, indicating similarity in material and energy consumption among the sublines.

Symbionts often affect host insect behavior. *Cardinium* affects the reproductive behavior of the host insect (Provencher et al. 2005). *Wolbachia* infection drastically suppresses a parasitic wasp (*Leptopilina heterotoma*) of *Drosphila* (Fleury et al. 2000). Endosymbionts can also change the probing behavior of host insects. *Wolbachia* adversely affects mosquito probing behavior (Moreira et al. 2009, Turley et al. 2009). The difference in the probing behavior of *B. tabaci Cardinium*-infected (C^+^) and *Cardinium*-uninfected (C^-^) sublines may be related to the difference in fitness (Zhang et al. 2016). The previous research has shown that the C^-^ subline has greater fitness than the C^*+^ subline. Thus, our hypothesis that *Cardinium* has a negative influence on the probing behavior of *B. tabaci* MED was supported. Whiteflies that lack the *Cardinium* endosymbiont ingest phloem sap for the longest time period are also likely to be the most fit.

That said, it is also possible that the gold wires we used had a negative effect on our whiteflies. Even the thinnest gold wires are highly encumbering for tiny whiteflies, and this encumbrance affects their recorded stylet probing behaviors. Most whitefly EPG studies use gold wire, as in our study, probably because it is relatively inexpensive and easy to purchase and to use. However, Chesnais et al. (2018) showed that gold wire tethers inhibit normal *B. tabaci* probing behavior by reducing a number of potential drops, disrupting access to the phloem, and reducing the ease of passive phloem ingestion (E2). The results of Chesnais et al. (2018) suggest that the lack of potential drops seen in our study may have resulted from the use of gold wire rather than thinner, platinum wire. If this study had used platinum rather than gold wire, *B. tabaci* could have shown more natural movement and probing behaviors, and the number of *B. tabaci* that reached the phloem of cotton may have increased. Whether these results would be consistent with the results of this experiment requires further experimentation. However, we speculate that our phloem ingestion results would be enhanced by better wire, and perhaps further significant differences would be found in potential drops and other aspects of the non-phloem phase.

This experiment explored the effect of *Cardinium* on the probing behavior of *B. tabaci* MED on the premise of eliminating the genetic background of the test insects. *Cardinium* interfered with the probing behavior of *B. tabaci* MED. Differences in probing behavior of the *B. tabaci* MED C^*+^ subline and C^-^ subline were caused by the presence of *Cardinium*. We suggest that other methods (such as gene fluorescence quantification and RNAi) be combined to verify the relationship between *Cardinium* and *B. tabaci* MED, and to analyze the fitness differences between whiteflies.

## Conclusion

This study provides information on the effects of symbiotic bacteria on whitefly feeding behavior. After eliminating genetic differences between whitefly sublines, the uninfected C^-^ subline ingested phloem more readily than the C^*+^ subline. This indicated the influence of *Cardinium* on the stylet probing behavior of *B. tabaci* MED. Understanding whitefly behavior with or without symbiotic bacteria may help improve whitefly pest management.
